# Supplementary material for: Saccharomyces boulardii (CNCM I-745) improves intestinal damage in sepsis by remodeling intestinal flora structure
Source: Front Cell Infect Microbiol. 2025 Jul 31;15:1602792. doi: 10.3389/fcimb.2025.1602792 (PMC12350329; doi:10.3389/fcimb.2025.1602792)
Supplement: Supplementary file 1 [file DataSheet1.zip › summary/summary/7_Differential_analysis/2_LEfSe_result/Group_CW4_vs_AW4_0.05_3.0/LEfSe_Group_CW4_vs_AW4.cladogram.pdf]

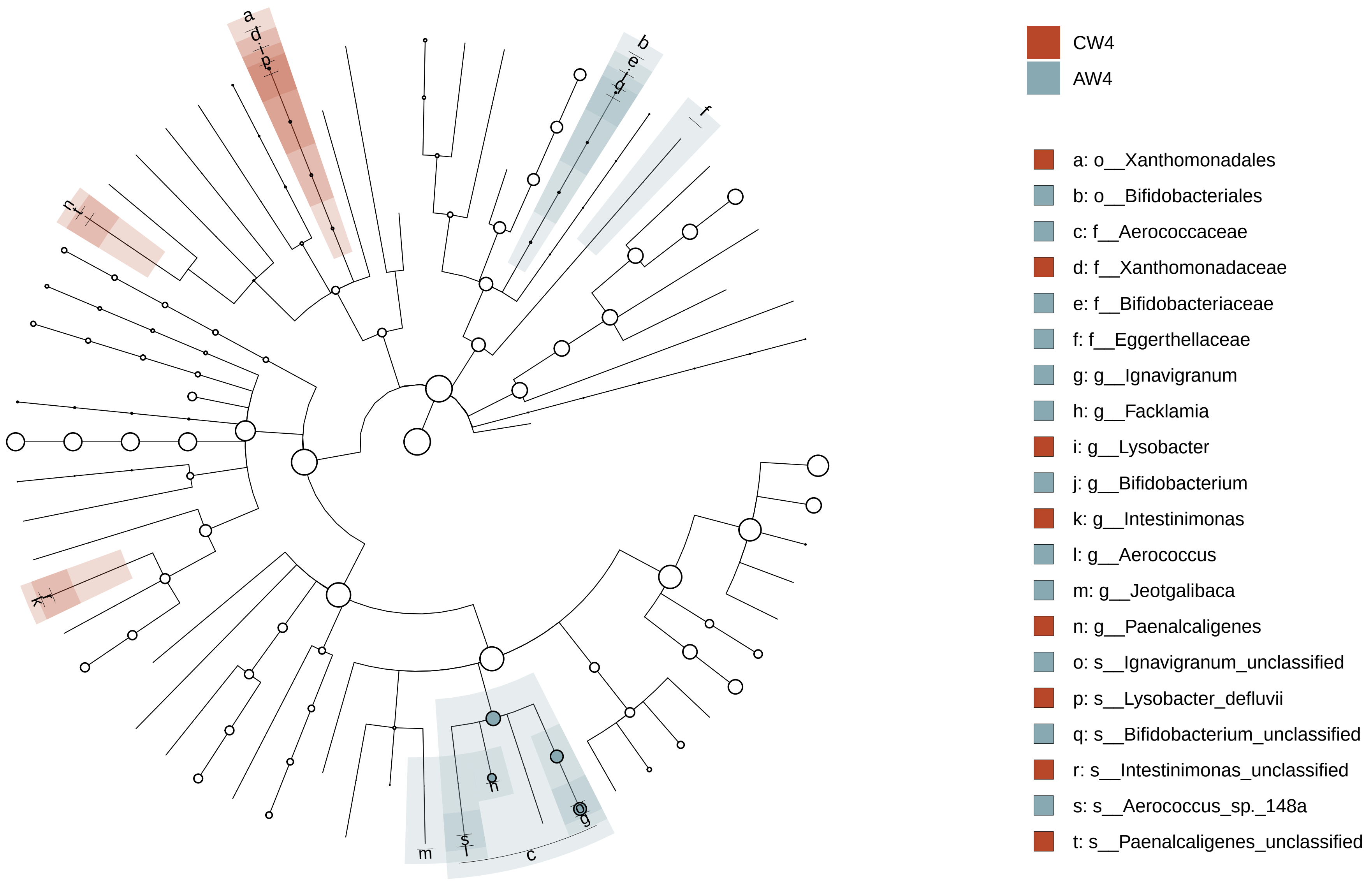
CW4
AW4
a: o__Xanthomonadales
b: o__Bifidobacteriales
c: f__Aerococcaceae
d: f__Xanthomonadaceae
e: f__Bifidobacteriaceae
f: f__Eggerthellaceae
g: g__Ignavigranum
h: g__Facklamia
i: g__Lysobacter
j: g__Bifidobacterium
k: g__Intestinimonas
l: g__Aerococcus
m: g__Jeotgalibaca
n: g__Paenalcaligenes
o: s__Ignavigranum_unclassified
p: s__Lysobacter_defluvii
q: s__Bifidobacterium_unclassified
r: s__Intestinimonas_unclassified
s: s__Aerococcus_sp._148a
t: s__Paenalcaligenes_unclassified
